# Immune Mechanisms Linking Obesity and Preeclampsia

**DOI:** 10.3390/biom5043142

**Published:** 2015-11-12

**Authors:** Frank T. Spradley, Ana C. Palei, Joey P. Granger

**Affiliations:** Department of Physiology and Biophysics, Cardiovascular-Renal Research Center, Women’s Health Research Center, The University of Mississippi Medical Center, Jackson, MS 39216, USA; E-Mails: fspradley@umc.edu (F.T.S.); apalei@umc.edu (A.C.P.)

**Keywords:** Adipose, antibody, endothelin, hypertension, lymphocyte, pregnancy

## Abstract

Preeclampsia (PE) is characterized by hypertension occurring after the twentieth week of pregnancy. It is a significant contributor to maternal and perinatal morbidity and mortality in developing countries and its pervasiveness is increasing within developed countries including the USA. However, the mechanisms mediating the pathogenesis of this maternal disorder and its rising prevalence are far from clear. A major theory with strong experimental evidence is that placental ischemia, resulting from inappropriate remodeling and widening of the maternal spiral arteries, stimulates the release of soluble factors from the ischemic placenta causing maternal endothelial dysfunction and hypertension. Aberrant maternal immune responses and inflammation have been implicated in each of these stages in the cascade leading to PE. Regarding the increased prevalence of this disease, it is becoming increasingly evident from epidemiological data that obesity, which is a state of chronic inflammation in itself, increases the risk for PE. Although the specific mechanisms whereby obesity increases the rate of PE are unclear, there are strong candidates including activated macrophages and natural killer cells within the uterus and placenta and activation in the periphery of T helper cells producing cytokines including TNF-α, IL-6 and IL-17 and the anti-angiogenic factor sFlt-1 and B cells producing the agonistic autoantibodies to the angiotensin type 1 receptor (AT1-aa). This review will focus on the immune mechanisms that have been implicated in the pathogenesis of hypertension in PE with an emphasis on the potential importance of inflammatory factors in the increased risk of developing PE in obese pregnancies.

## 1. Introduction

Preeclampsia (PE) is a complex maternal syndrome characterized by multiple disorders including proteinuria, thrombocytopenia, renal insufficiency, impaired liver function, pulmonary edema and cerebral or visual symptoms, but is most identifiable by the development of new-onset hypertension (systolic blood pressure ≥140/diastolic blood pressure ≥90 mmHg) after the twentieth week of gestation [1]. According to the PE Foundation’s website, PE is responsible for as many as 100,000 in 500,000 preterm births in the United States (PE Foundation. 17 January 2014. A Day the World Pauses for Premature Babies. Retrieved from http://www.PE.org/component/content/article/3-newsflash/219-a-day-the-world-pauses-for-premature-babies). Thus, PE is a significant contributor to the estimated 15 million preterm births annually worldwide [2]. It is a leading cause of maternal and perinatal morbidity and mortality [3,4].

To understand the mechanisms that promote the development of PE, it is first important to know how a healthy pregnancy is established and maintained. A healthy pregnancy is dependent on the ability of the maternal cardiovascular system to adapt to the needs of the growing utero-placental-fetal unit. This is aided by widespread maternal vasodilation in the periphery and the uterus to accompany the approximate doubling of blood volume that occurs throughout gestation to allow blood and necessary nutrients to reach the growing fetus and placenta [5,6]. During normal pregnancy, fetally-derived cytotrophoblast cells migrate through the endometrium and myometrium where they invade and replace the smooth muscle and endothelial cells of the uterine spiral arteries. There they remodel these arteries from small diameter, high-resistance vessels to larger and more capacitance vessels [7]. However, when these vasodilation and vascular remodeling processes are not sufficient, pregnancy complications can ensue [8,9]. Indeed, a prevailing theory behind the etiology of PE is that mechanisms that promote utero-placental vascular remodeling during a healthy pregnancy are impaired, which can result in placental ischemia [10]. Evidence from mice and *in vitro* models has demonstrated that reduced migration and invasion of cytotrophblast cells into the maternal uterus prohibits uterine spiral artery remodeling and widening [11,12]. Moreover, studies utilizing placental sections taken from PE women have found a significant correlation between reduced utero-placenta vascular remodeling and attenuated invasion of cytotrophoblast cells into these blood vessels [13].

Intensive research efforts have been directed toward examining the mechanisms that mediate peripheral and uterine vascular remodeling during healthy pregnancy and how these pathways are altered during PE leading to placental ischemia. Recent evidence has indicated that aberrant immune cell and cytokine signaling result in improper cytotrophoblast proliferation, migration and invasion. Resident uterine natural killer (uNK) cells, which are the most abundant leukocytes in early human and mouse decidua, regulate invasion of trophoblast cells and uterine vascular remodeling and angiogenesis. This was demonstrated in experiments where adoptive transfer of NK cells from Rag2^−/−^ mice (having NK cells but not T or B lymphocytes) into BALB/c-Rag2^−/−^Il2^−/−^ mice (having no NK, T or B cells) fully reversed the uterine vascular defects found in the latter mouse [14]. Extravillous trophoblast (EVT) cells are derived from cytotrophoblast cells that compose the cell columns anchoring and attaching the placenta to the uterine wall [15]. It is these EVT cells that are hypothesized to invade the uterus and prompt uterine vascular remodeling [16]. Co-culture experiments of human uNK and EVT cells with isolated spiral arteries demonstrated that uNK cells are important for the process of spiral artery remodeling [17].

In contrast, it has been observed in human placenta that the lack of invasiveness of EVT cells is accompanied by over activation of pro-inflammatory immune cells with increased macrophage counts. *In vitro* studies confirmed that activated macrophages inhibit cytotrophoblast migration [18]. As a result of reduced placental vascular remodeling and uterine blood flow, placental ischemia and placental inflammation occur leading to release of numerous factors from the ischemic placenta into the maternal bloodstream. There they activate peripheral immune cells including T and B lymphocytes mediating targeted endothelial cell and vascular dysfunction and hypertension [19].

Thus, PE is thought to occur in two stages. The first stage involves cytotrophoblast dysfunction with failure of utero-placental vascular remodeling and widening leading to placental ischemia. This is accompanied by molecular changes such as increased levels of hypoxia-inducible factor (HIF)-1α in the human placenta and several animal models of surgically-induced placental ischemia in monkeys, rats and mice [20]. Knockdown of HIF-1α using siRNA technology attenuated hypertension and proteinuria induced by inflammatory factors, namely, agonistic autoantibodies to the angiotensin II type 1 receptor (AT1-aa) or tumor necrosis factor superfamily member 14 (LIGHT) in mice [21]. The second stage involves the release of vasoactive factors from the ischemic placenta into the maternal circulation where they promote endothelial and vascular dysfunction bringing about maternal vasoconstriction, increasing total peripheral resistance and reducing renal excretory function to promote hypertension [22,23,24,25]. The first goal of this review is to detail the importance of proper immune system function for the maintenance of a healthy pregnancy for the purpose of discussing how inappropriate activation of this system promotes the pathogenesis of PE. Our second goal is to propose that an exaggerated activation of the immune system may be an important mechanism linking obesity with increased risk of developing PE.

## 2. The Prevalence of PE and Obesity Are on the Rise

**Figure 1:**
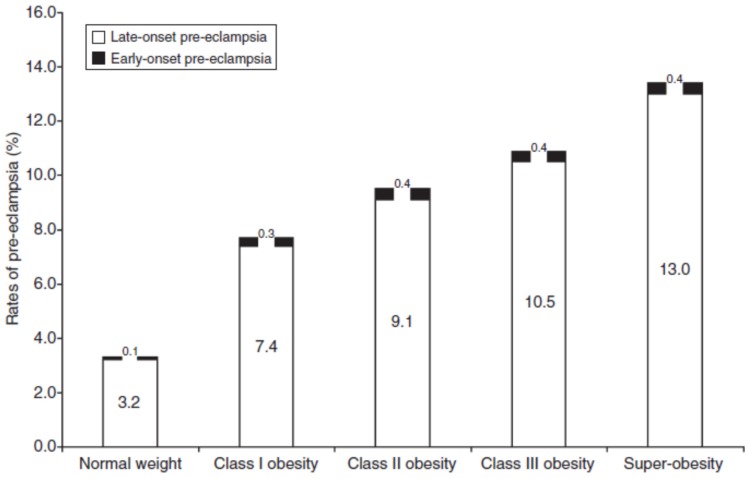
Increasing obesity increases the risk for PE. The incidence of PE is 3% in normal weight gravidas (BMI = 18.5–24.9), 7% in those gravidas with class I obesity (BMI = 30–34.9), 9% with class II (BMI = 35–39.9), 11% with class III obesity (BMI = 40–49.9), 13% in super-obese women (BMI = 50). From: Mbah, A.K., *et al*. [31].

A cohort of datasets assembled by the US Centers for Disease Control and Prevention, which included 120 million women admitted to hospital for delivery from all 50 states and the District of Columbia, demonstrated that the rate of PE increased from 3.4% to 3.8% between the years 1980–2010 [26]. Although this may not seem like a dramatic increase, it was noted that whereas the occurrence of mild PE (SBP: 140–159/DBP: 90–109 mmHg) has declined by 19% from 3.1% to 2.5%, the increase in the overall prevalence was due to a 322% increase in the severe form of PE (SBP: ≥160/DBP: ≥110 mmHg). This study further concluded that obesity was a major driving factor for the increase in this maternal disorder. Other studies have also indicated that the increasing rate of PE is most likely due to the concurrent increase in obesity [27,28], which has reached pandemic proportions. Even low- and middle-income countries are experiencing increased obesity [29]. Since the early 1960s, obesity among adults aged 20 years or older has risen from 13.4 to 35.7 percent in the USA [30]. This increased prevalence of obesity during the reproductive years has lead researchers to find that there is a stepwise increase in the rates of PE with increasing body mass index (BMI) class [31] (Figure 1). However, the mechanisms linking obesity and increased risk for PE are not yet clear.

**Figure 2:**
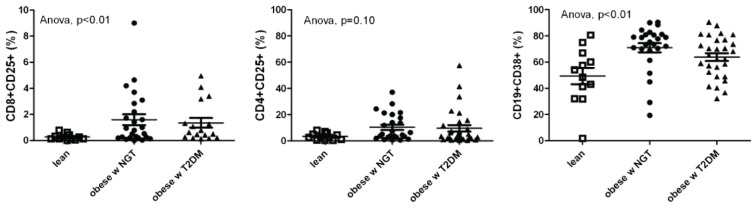
CD8^+^CD25^+^ cytotoxic T cells (as % of CD8, left), CD4^+^CD25^+^ T helper cells (as % of CD8, middle) and CD19^+^CD38^+^ B cells (as % of CD19 cells, right) in non-pregnant lean and obese women with normal glucose tolerance (NGT) or type-2 diabetes mellitus (T2DM). From: van Beek, L., *et al*. [35].

Obesity is a state of chronic, low-grade inflammation [32,33]. As it has been shown that pro-inflammatory mechanisms mediate the development of placental ischemia-induced hypertension, it is reasonable to hypothesize that these mechanisms are exaggerated in the obese state. This could be an explanation as to why obese pregnant women are at increased risk for PE. Interestingly, obese non-pregnant women of reproductive age have increased circulating CD4^+^, CD8^+^ and B lymphocyte counts (Figure 2) and also total lymphocyte and white blood cell counts [34,35]. In addition, it has been shown that mononuclear cells isolated from these women are unable to suppress pro-atherogenic inflammation such as production of interleukin (IL)-6, IL-1β, reactive oxygen species (ROS) and the pro-inflammatory transcription factor NF-κB in response to hyperglycemia [36]. This has been recapitulated in animal models. For example, high-fat diet feeding in non-pregnant female mice led to increased body weight, white adipose tissue mass and hypertrophy, hyperinsulinemia, hyperglycemia and increased adipose tissue inflammation with accumulation of macrophages [37]. The bone marrow from these high-fat diet-fed female mice is primed to make more activated myeloid progenitors cells. These investigators found that saturated fatty acids are able to produce this effect in bone marrow isolated from female mice. Separate studies have revealed that, under non-pregnant conditions, overweight and obese women have endothelial dysfunction assessed by flow-mediated vasodilation in the brachial artery [38], and non-pregnant apoE knockout female mice having metabolic derangements including elevated circulating cholesterol also have endothelial dysfunction [39,40].

**Figure 3:**
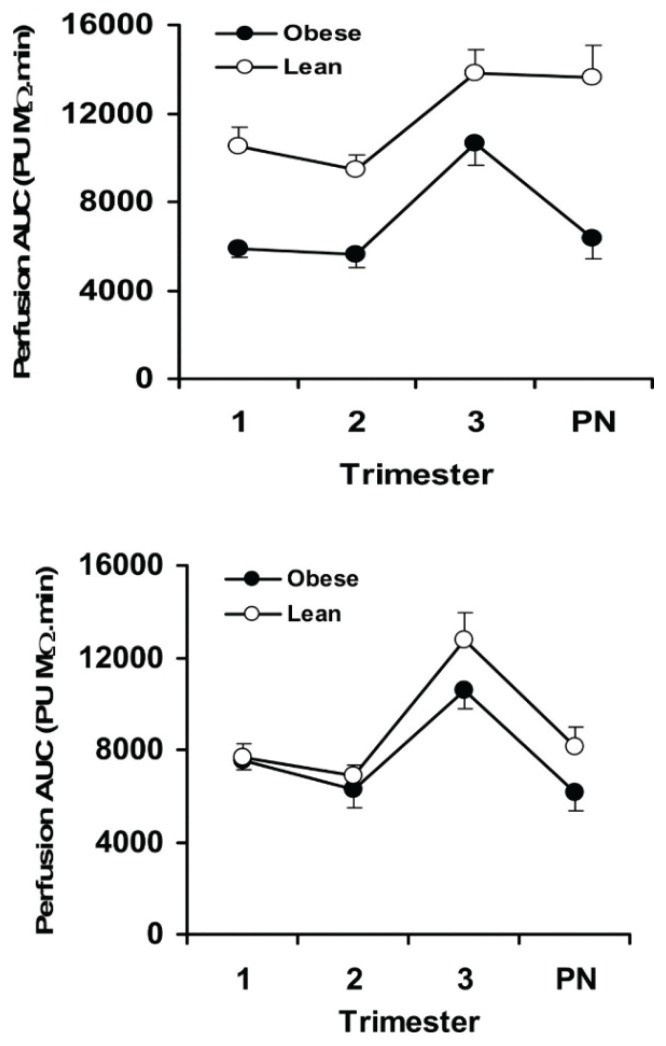
Upper panel: Endothelial-dependent vasodilation in response to iontophoresis of ACh in pregnant lean (*N* = 30) versus obese (*N* = 30) women measured at each trimester and at least 4 weeks postnatal (PN). Overall response was significantly greater in lean versus obese, *p* < 0.001. Lower panel: Endothelial-independent vasodilation in response to iontophoresis of SNP in pregnant lean women (*N* = 30) versus obese women (*N* = 30). Overall, there was a small but significant difference (*p* = 0.021) between the two groups. Mean ± SEM. From: Stewart, F.M., *et al*. [42].

With regards to the pregnant state, Wistar rats maintained on a 62% versus 12% fat diet from conception to gestational day 20 had increased circulating LDL cholesterol and triglycerides along with elevated tail-cuff systolic blood pressure [41]. Obese pregnant women present with reduced vasorelaxation responses to acetylcholine and sodium nitroprusside demonstrating attenuated endothelial-dependent and independent responses, respectively, throughout most of gestation in skin blood vessels (Figure 3), which may have been mediated by the increases in IL-6, C reactive protein, sICAM-1, PAI-1 and PAI-2 [42]. Myometrial arteries isolated from obese pregnant women at the end of pregnancy had reduced vasorelaxation responses as well [43]. Placental ischemia was not assessed in these studies, nor were the exact blood pressure values reported or markers of placental ischemia examined. However, these obese pregnant women did have endothelial dysfunction and reduced responsiveness to a nitric oxide (NO) donor, which suggests that they are predisposed to exaggerated placental ischemia-induced vascular dysfunction and hypertension.

Vascular dysfunction is a major downstream effect of placental ischemia resulting in reduced renal excretory function and the presentation of hypertension in PE. There is solid evidence that activation of the immune system, with the most widely studied effector cells being T and B lymphocytes, mediates placental ischemia-induced hypertension [19]. This inflammatory response to placental ischemia may be exaggerated in women with obesity due to the presence of obesity-related metabolic factors. Our goal is to propose novel immune mechanisms whereby obesity and obesity-related metabolic factors increase the risk for developing PE by: (1) promoting the development of placental ischemia; (2) exaggerating placental ischemia-induced inflammatory responses; and (3) sensitizing the maternal vasculature to inflammatory cytokines.

## 3. Obesity Increases Placental Inflammatory Cytokines, Immune Cells and Ischemia

Before discussing pathologies in obese PE pregnancies, it seems fitting to discuss immune mechanisms in healthy pregnancy. During pregnancy, the mother is exposed to foreign antigens derived from the sperm, fetus, and placenta. Therefore, the mother mounts an appropriate immune response to recognize these antigens as “self” to allow for immune tolerance and to avoid rejection of the pregnancy. Notably, PE is associated with primiparity as epidemiological studies have found that a previous healthy pregnancy or abortion (spontaneous or induced) reduces the risk of developing PE in subsequent pregnancies, although this protective effect is lost with a change of partner [44,45,46]. Human and animal data showed that seminal vesicle-derived transforming growth factor β1 (TGFβ1) initiates a type 2 immune response in the female reproductive system in order to allow sampling and processing of paternal antigens [47]. By stimulating a type 2 immune response, instead a type 1, seminal TGFβ1 may inhibit the development of the inflammatory reaction against the semi-allogenic fetus that is thought to be linked with poor placentation and PE. How obesity impacts on these immune responses and cytokine production is unknown and represents an important area of investigation.

### 3.1. Inflammatory Cytokines

**Figure 4:**
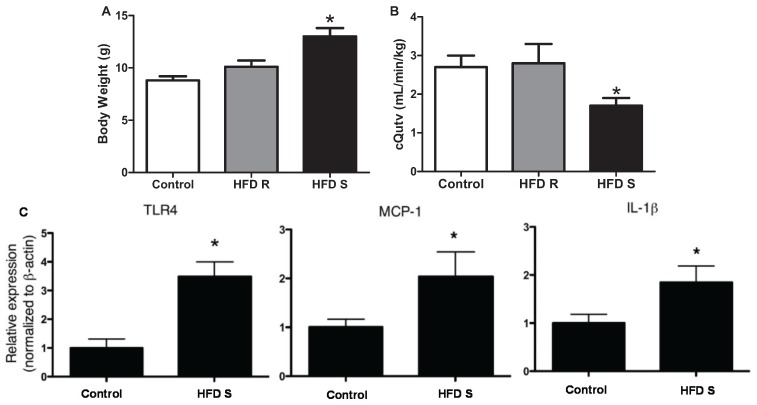
High-fat diet (HFD)-induced obesity (**A**) elicits reductions in placental volume blood flow (**B**), which is accompanied by increased placental gene expression of TLR-4, MCP-1 and IL-1β (**C**) in the third trimester of baboons. Control = control diet; HFD resistant (R) and HFD sensitive (S). * *p* < 0.05 *vs.* control and HFD R. From Frias, A.E., *et al*. [53].

Interestingly, increased BMI has been associated with activation of inflammatory pathways within the human placenta [48,49]. A recent study found that pre-pregnancy obesity was associated with increased expression of placental pro-inflammatory cytokines and circulating IL-6 by the end of pregnancy. In addition, there was a greater degree of muscularity in the vessel walls of obese compared to non-obese placentas [50]. Evidence for altered trophoblastic invasion and vessel remodeling were also observed in animal models of lifelong obesity where rats fed a high-fat diet from 3 to 19 weeks of age presented higher levels of smooth muscle actin (SMA) surrounding the placental spiral arteries on gestational day (GD) 18 [51]. Downstream in the labyrinth layer, there was increased carbonic anhydrase staining (a marker of hypoxia) in this placental compartment and elevated blood pressure in high fat diet-treated rats compared with control diet-treated rats on GD 15 [52]. Moreover, female monkeys that presented with increased body weight in response to 4 years of high-fat diet feeding (Figure 4A) showed reduced placental volume blood flow (Figure 4B) accompanied by increased placental infarction and calcification in the end of pregnancy compared to monkeys on control chow and those that did not have a significant increase in body weight following the high-fat diet regimen. The heavier, high-fat diet-treated monkeys presented a higher degree of inflammation within the placenta detected by increased gene expression of toll-like receptor (TLR)-4, monocyte chemoattractant protein (MCP)-1 and IL-1β, compared with control diet-treated pregnant monkeys (Figure 4C) [53].

It has been consistently demonstrated that the obesity-related metabolic factor leptin is elevated in the circulation of pregnant women destined to develop PE [54,55,56,57,58,59]. Gene and protein expressions of leptin are upregulated in PE placentas compared with normal placentas [60,61,62]. Those overweight/obese women with increased leptin concentrations are more likely to experience PE [55,56,59]. A recent report indicated that endothelial cells are the source for elevated leptin in placentas from women with increased pre-pregnancy BMI, but blood pressure was not examined in this study [63]. Similarly, leptin levels were increased in whole placental tissue collected at GD 21 from rats fed a saturated fat-rich diet from 6 to 16 weeks of age [64].

**Figure 5:**
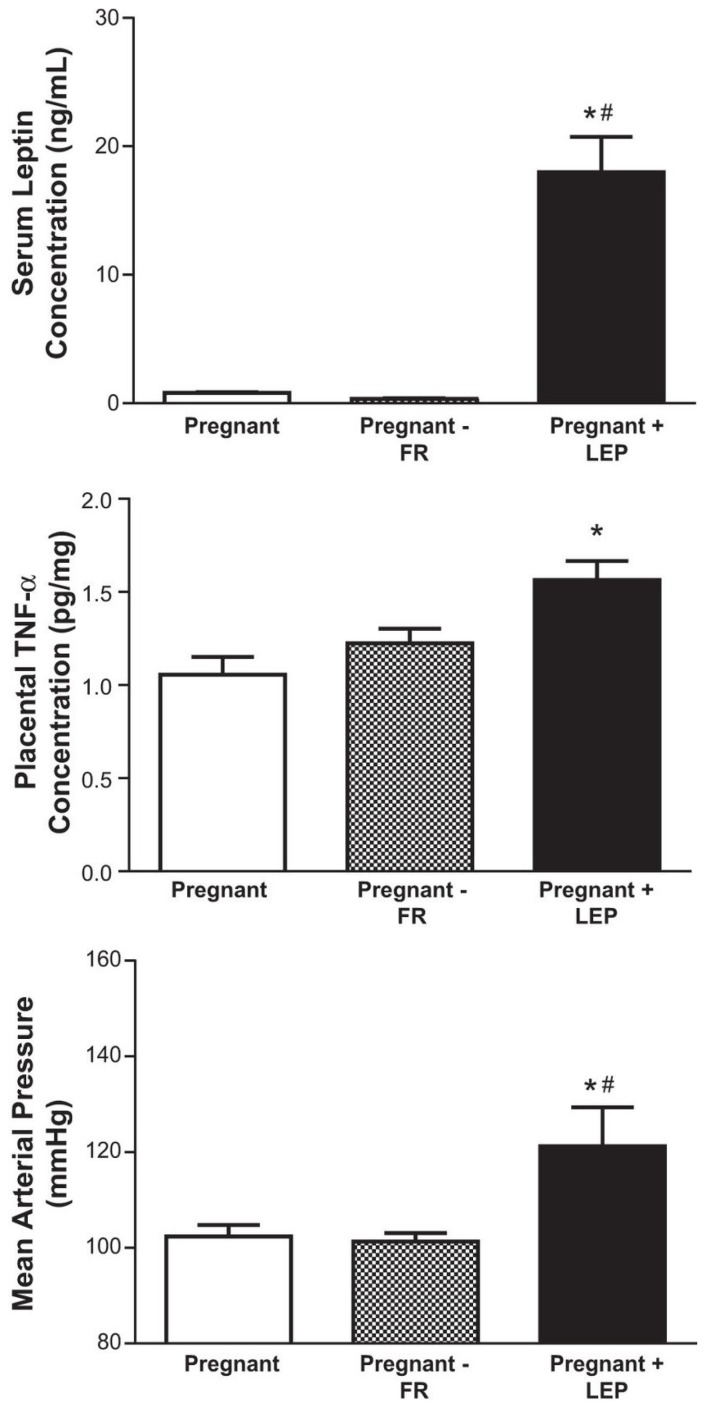
Leptin infusion (0.5 µg/kg/day) into pregnant rats (pregnant + LEP) from gestational day 14–19 elicits increases in circulating leptin (upper panel), mean arterial blood pressure (middle panel) and placental TNF-α levels (lower panel). A group of non-infused pregnant rats were food restricted (pregnant-FR) to match the reductions in food intake induced by leptin infusion. * *p* < 0.05 *vs.* pregnant; ^#^ *p* < 0.05 *vs.* pregnant-FR.

Intriguingly, placental leptin and HIF-1α mRNA levels [60] as well as circulating leptin and TNF-α are positively correlated in human PE [65] and also placental TNF-α in pregnant rats [66]. Leptin secretion by BeWo cells is increased when cultured under hypoxia (5% O_2_) compared with those cells cultured under normoxic conditions (20% O_2_) [62], and leptin induces the release of TNF-α from human placental and adipose explants [67]. These studies, plus our findings that chronic hyperleptinemia leads to hypertension in pregnant rats and increases TNF-α levels in their placentas, which occurred independently of leptin-induced reductions in food intake as determined by food-restricting normal pregnant rats to match the food intake of those receiving leptin (Figure 5) [66], point to leptin as a link between obesity and PE. We hypothesize that additional obesity-related metabolic factors may also play a similar or even synergistic pro-inflammatory role in the placenta as *in vitro* studies have demonstrated that treatment of human choriocarcinoma BeWo trophoblast cells with palmitic acid, which is the most abundant circulating free fatty acid in obesity, in combination with TNF-α led to feed-forward production of both TNF-α along with increases in IL-6 [68].

### 3.2. Immune Cells

During the first weeks of a normal gestation, there is an increase in the number of natural killer (NK) cells and macrophages at the maternal-fetal interface. In addition to having a local immune function, these native immune cells are critical for placental organogenesis by promoting trophoblast recruitment, spiral artery remodeling, and angiogenesis. Moreover, NK cells and macrophages regulate trophoblastic invasion and tissue remodeling by producing a broad variety of cytokines, chemokines, angiogenic factors, and proteases [69,70]. Interestingly, altered decidual leukocyte populations have been described in PE [71], suggesting that shallow trophoblastic invasion and abnormal spiral artery remodeling may be a result of an inappropriate immune cell response. This section will discuss studies supporting that aberrant immune system activation and inflammation leads to abnormal placental development. Because they represent the highest populations in the first trimester human decidua, we will focus on altered number and function of NK cells and macrophages as pathophysiological mechanisms linking obesity to placental ischemia.

#### Uterine Natural Killer Cells (uNKs)

An adequate and orchestrated interaction of fetal trophoblasts and maternal cells present in the decidua is required for a successful placentation. The uNK cells compromise ~70% of the leukocyte population in early human pregnant decidua [72]. Although uNK cells have a prominent role in cytokine secretion, rather than immune defense, they do express proteins with cytolytic capacity to fight infections [73]. However, through the interaction between natural cytotoxicity receptors and killer cell immunoglobulin-like receptors (KIR)s in NK cells and major histocompatibility complex (MHC) molecules in fetal trophoblasts, the cytotoxic machinery of uNK cells is not activated to respond against human EVT cells invading the uterine tissue [74,75,76].

As spiral artery remodeling takes place before the appearance of EVTs, it has been suggested that uNK cells may initiate and aid EVTs to then complete this process [77]. The first studies demonstrating the importance of uNK in pregnancy were performed in transgenic mice obtained by gene ablation and crossing homozygous NK and T cell-deficient tg epsilon 26 (Tge26) mice, which have less than 1% of normal uNK cell number; no development of implantation site-associated metrial gland; edematous deciduas with abnormally high vessel walls/lumens ratios; small placentas; and partial fetal loss [78]. Transplantation of bone marrow from B and T cell-deficient scid/scid donors into Tge26 mice restored uNK cell population; induced metrial gland differentiation; reduced decidual abnormalities; and increased placental sizes and fetal viability during gestation [79]. Transplantation of bone marrow from mice possessing NK cells unable to produce either interferon (IFN)-γ, components of the IFN-γ signaling pathway, or the IFN-γ receptor into completely NK cell-deficient mice resulted in decidual disorganization and altered vessel modification during pregnancy [80]. Treatment of these NK cell-deficient mice with IFN-γ restored decidual morphology [81].

Immunohistochemical studies with serial sections of human placentas in the first trimester showed that uNK cells (identified by CD56 positivity) infiltrate spiral arteries prior to trophoblast cells. This occurs during a stage of active vessel remodeling characterized by disruption of smooth muscle vascular cells (SVMCs) and breaks in the endothelial lining with these cells undergoing apoptosis resulting in disorganization of vascular layers [82]. Moreover, it has been demonstrated that human uNK cells secrete inflammatory factors, such IL-8, IFN-γ and TNF-α, and vasoactive factors, such as angiopoietin (Ang)-1, Ang-2 and vascular endothelial growth factor (VEGF)-C, which promote apoptosis [17,83,84,85]. These factors can also serve as signals from uNK cells to EVTs inducing the latter’s migration and differentiation. For example, IL-8 stimulates recruitment and migration of EVTs to spiral arteries [74]. On the other hand, TNF-α alone or in combination with IFN-γ inhibits EVT invasion by increasing trophoblast apoptosis and decreasing trophoblast proliferation. While TNF-α alone promoted these effects in EVT cells through stimulation of pro-matrix metalloproteinase (MMP)-9 (but not active MMP-9), urokinase plasminogen activator (uPA) and plasminogen activator inhibitor (PAI)-1 levels, the combination of TNF-α and IFN-γ induced a reduction in pro-MMP-2 and an increase in uPA [86]. Yet, IL-8 increases expression of integrins α1 and β5 and up-regulates MMP-2 and MMP-9, favoring the EVT differentiation into an invasive phenotype [87]. Conversely, VEGF-C favors the differentiation of EVTs into an endovascular phenotype by stimulating their formation into capillary tubes [84]. Taken together, these last studies suggest that the cytokine milieu secreted by NK cells is critical in determining regional and temporal changes in EVT invasion.

Obesity may promote deleterious interactions between uNK cells and trophoblasts. Although this issue has not yet been addressed in pregnant humans, Parker *et al*. [88] showed that high-fat/sugar diet-induced obesity reduces uterine NK cell number and their expression of IFN-γ in pregnant mice. Intriguingly, non-pregnant obese patients have less circulating CD56^+^ NK cells than lean controls, with these numbers being even lower in metabolically unhealthy obese patients [89]. *In vitro* studies have shown that adipocyte-conditioned medium is able to increase expression of the cytotoxic enzyme granzyme A in human peripheral blood CD56^bright^ NK cells [90]. Obesity is linked to over-activation of inflammatory processes within the placenta [48,50]. Interestingly, leptin in doses comparable to those achieved during the first and third trimesters of gestation can act as a pro-inflammatory cytokine upregulating the production of TNF-α by peripheral blood mononuclear leukocytes. In addition, first and second trimester concentrations of leptin evoked different CD56 and CD16 expressions in the membrane of these cells [91]. Curiously, both peripheral blood CD56^bright^ and CD56^dim^ NK cells express leptin receptors and, while short-term (up to 24 h) leptin incubation caused an increase in IFN-γ and cytotoxic activity of these cells, long-term (more than 4 days) leptin exposure significantly impaired NK cell immune function and decreased cell proliferation [92]. Likewise, other reports have described a role for obesity and leptin in regulating circulating NK cells, but authors have identified their NK cell population by other ways than the presence or absence of the CD56 marker [93,94,95,96].

### 3.3. Macrophages

In normal pregnant women, macrophages compromise ~20% of the decidual leukocyte population. In contrast with uNK cells, macrophage number does not change substantially with increasing gestational age, although the ratio of macrophages increases to NK cell number increases in uterine tissue during the second half of pregnancy [72]. Uterine macrophages are important for protecting the fetus against maternal immune intolerance [97] and infectious agents [98]. However, because leukocytes are present in decidua when disruption and disorganization of VSMCs and endothelial cells is active before the arrival of trophoblasts [82], a role for macrophages in spiral artery remodeling has been suggested. Indeed, macrophages (identified by CD14 or CD68 positivity) are usually found in close proximity to spiral arteries and uterine glands, as well as associated with EVTs [72]. In addition, it has been shown that macrophages secrete many cytokines, angiogenic factors, and proteases [99]. Moreover, macrophages clear apoptotic trophoblasts and other apoptotic bodies, preventing the release of pro-inflammatory molecules from these cells into the decidua [100]. Collectively, these studies provide evidence for the relevance of proper macrophage function during the early phases of vascular remodeling.

Although some reports showed a decreased macrophage number in third trimester decidua of PE patients compared with pregnant controls [71,101], others have found an increased macrophage number [102,103]. As mentioned before, conflicting findings across studies may be explained by the use of different cell makers or methodologies. Yet differences in macrophage numbers may only be regional, because the immunohistochemistry stain was noted to be higher specifically around spiral arteries in PE [102]. Increased number of decidual macrophages is in line with the evidence of elevated levels of macrophage chemotactic factors, such as M-CSF and MCP-1, in PE [104,105]. Nonetheless, because levels of inflammatory and anti-inflammatory cytokines are increased and decreased, respectively, in PE placentas [106], it is supposed that decidual macrophages are differently activated in PE. Indeed, *in vitro* studies have demonstrated that only activated macrophages (by e.g., TNF-α) are able to limit trophoblastic invasion of spiral arteries through apoptosis [18,107]. Intriguingly, recent reports indicate that decidual macrophages express the Flt-1 receptor [103,108]. While first and third trimester macrophages isolated from the placenta naturally secreted low levels of sFlt-1, under lipopolysaccharide stimulation the sFlt-1 release was 4-fold increased [108].

It has been also shown that macrophages differ phenotypically in PE in a way that the CD163^+^/CD14^+^ and CD206^+^/CD68^+^ ratios were reduced in decidual tissue of PE patients and pregnant women destined to develop PE, respectively [103,109]. Additionally, decreased number of CD206^+^ macrophage has been found in uterine tissue of a rat model of PE on GD 20 [110]. Therefore, it has been suggested that a deviation from this M2 immunoregulatory type to the M1 pro-inflammatory type occurs in PE [69,111]. Interestingly, *in vitro* studies have demonstrated that excess of macrophage-derived TNF-α enhances expression of MMP-1, MMP-3 and MMP-9 in decidual cells, interfering with the normal stepwise process of trophoblastic invasion [112]. Because M2 macrophages are the ones producing the proteases required to degrade the extracellular matrix surrounding spiral arteries [113], reduced M2 cell number in the decidua might also lead to impaired vascular remodeling.

**Figure 6:**
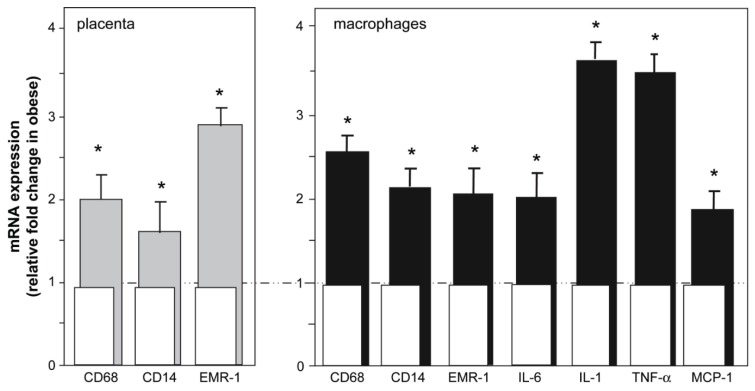
(**Left**) mRNA expression of macrophage markers in whole placenta villous tissue from obese (**grey bars**) and lean women (**white bars**). (**Right**) mRNA expression of inflammatory markers in isolated placental CD14+ macrophages in obese (**black bars**) and lean women (white bars). Mean ± SEM. From: Challier, J.C., *et al*. [114]. Expression values in lean women were set at one to assess fold change from this value in obese women. * *p* < 0.001 on obese compared to lean women.

In humans, maternal obesity increases placental production of MCP-1, which is accompanied by an increased accumulation of macrophages in placenta [50,114], and macrophages isolated from these obese placentas produce greater number of cytokines including IL-1 and TNF-α (Figure 6). Likewise, studies in experimental models have found that consumption of obesogenic diets increases placental mRNA levels of MCP-1, CD14^+^ and CD68^+^ and placental macrophage infiltration [53,115,116]. Intriguingly, *in vivo* and *in vitro* studies from Benyo *et al*. [117] suggest that sources other than the placenta contribute to the elevated TNF-α and IL-6 concentrations observed in the circulation of PE patients. In fact, circulating monocytes isolated from PE patients produce greater TNF-α and ROS than cells of normal pregnant women [118]. In obese pregnant women, at least, it was noted that circulating and placental CD14^+^ monocytes share remarkable phenotypic and genotypic similarities. However, the expression of genes related to immune sensing and regulation, lipid metabolism, and extracellular matrix remodeling were increased 2–2006 fold in placenta compared with blood monocytes, implying that both sources may contribute to the propagation of inflammation at the maternal-fetal interface [119].

Finally, it has been shown that maternal obesity can lead to a lipotoxic placental environment characterized by decreased angiogenic regulators and increased inflammatory and oxidative stress markers [49]. This detrimental environment might favor the development of acute atherosis in spiral arteries, which consist of an arterial lesions depicted histologically by fibrinoid necrosis of vessel walls with foam cell infiltration [120]. As in atherosclerosis, it has been demonstrated that these foam cells have macrophage origins. While in atherosclerosis there is accumulation of foam cells and migration and proliferation of smooth muscle cells within the lesion, in acute atherosis the endothelial cells remain in the lesion and the smooth muscle layer surrounding the aggregates of fibrin and foam cells become thin or completely damaged [105]. Acute atherosis is mainly observed in poorly remodeled spiral arteries, and recent reports have described an association between the presence of placental acute atherosis and PE [105,121]. In view of these common immune and inflammatory findings leading to impaired placental function in obesity and PE, we propose that decreased decidual NK cell, increased macrophage infiltration, and altered activation of these cells are underlying etiological mechanisms that link obesity with placental ischemia.

## 4. Effects of Obesity on Placental Ischemia-Induced Peripheral Inflammation, Vascular Dysfunction and Hypertension

Studies in humans and animal models have indicated that the development of hypertension in PE has a strong pro-inflammatory component and that placental ischemia is the stimulus for this immune activation [19]. Most noted is activation of the adaptive immune system, namely T and B lymphocyte cells. The ischemic placenta induces activation of peripheral T cells, which can directly promote the development of hypertension by production of cytokines such as TNF-α and IL-6 and the anti-angiogenic factor sFlt-1 and by stimulating B cells to produce AT1-aa. Thus, T and B cells seem to be major effector immune cells whereby placental ischemia induces vascular dysfunction and hypertension. In this section, we will discuss the effects of obesity on placental ischemia-induced peripheral T and B cell activation; vascular inflammation and dysfunction; and the vasoconstrictor peptide endothelin (ET)-1 as a mechanism linking obesity and inflammation to vascular dysfunction and hypertension in PE.

### 4.1. T lymphocytes

**Figure 7:**
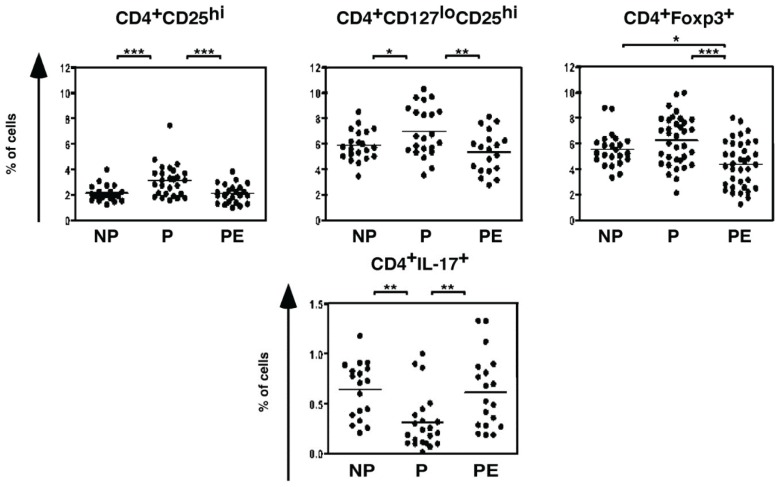
There are reduced suppressive CD4^+^CD25^hi^ (upper panel, left), CD4^+^CD127^lo^CD25^hi^ (upper panel, middle) and CD4^+^Foxp3^+^ (upper panel, right) T regulatory cells but increased pro-inflammatory CD4^+^IL-17^+^ T helper cells (lower panel) in PE women. Blood was collected during the third trimester from pregnant (P) and PE women (PE) and from age-matched non-pregnant women (NP). * *p* < 0.05, ** *p* < 0.01 and *** *p* < 0.0001. From: Santner-Nanan, B., *et al*. [125].

T lymphocyte cells are a critical arm of the adaptive immune response, which play a central role in cell-mediated immunity [122]. There are reduced CD4^+^CD25^hi^, CD4^+^CD127^lo^CD25^hi^ and CD4^+^foxp3^+^ T regulatory cells, which are known to control and prevent autoimmune disease [123,124], and increased pro-inflammatory CD4^+^IL-17^+^ T helper cells in PE women [125] (Figure 7). A more defined role for this immune cell type in mediating the hypertension in response to placental ischemia was confirmed in pregnant rats with reduced uterine perfusion pressure (RUPP). This is an experimental model of placental ischemia where silver clips are placed on the abdominal aorta above the uterine arteries and on the arcade of ovarian arteries leading to the first fetus of each uterine horn [126]. This surgical procedure, which is performed on gestational day 14, reduces uterine blood flow by ~40% and elicits dramatic hypertension and intrauterine growth restriction when assessed on day 19 and increased uterine artery resistive index [25,127]. The hypertension in this model is accompanied by increased activation of CD4^+^ T helper cells [128,129,130].

Circulating populations of pro-inflammatory immune cells are found to be greater in obese versus normal weight pregnant women [34,35]. Although during normal pregnancy there is reduced T cell activation [131], studies suggest in obese pregnancies that activation of CD4^+^ T cells is an impending mechanism that exacerbates placental ischemia-induced hypertension. It has been shown that dietary fatty acids influence the production of Th1- but not Th2-type cytokines [132]. Specifically, it was demonstrated that an increase in the balance between saturated over polyunsaturated fat promotes the production of pro-inflammatory cytokines. Serum concentrations of lipids have been shown to rise during the second and third trimesters when the complications of PE arise. Indeed, Robert’s group showed that obese women who develop PE have higher levels of triglycerides in the second trimester and free fatty acids in their third trimester [133].

**Figure 8:**
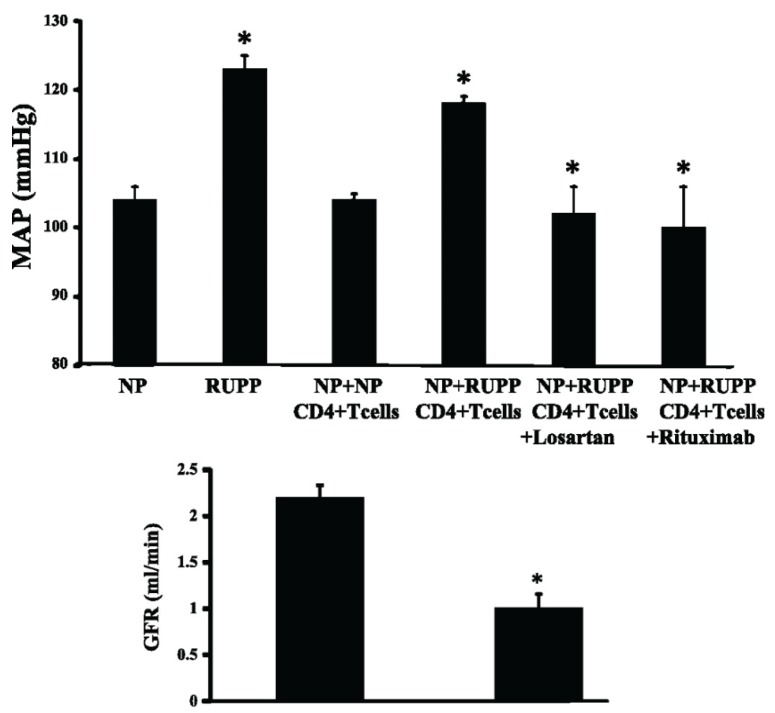
**Upper:** Mean arterial blood pressure (MAP) in: normal pregnant (NP, *N* = 10) rats; reduced uterine perfusion pressure rats (RUPP, *N* = 20); adoptive transfer of RUPP CD4+ T cells into NP recipient rats (*N* = 11); NP recipient rats of NP CD4+ T cell (*N* = 5); rats chronically-administered with the AT1R antagonist losartan; and rats with B cell depletion using rituximab (*N* = 4). **Lower:** Glomerular filtration rate (GFR) in NP rats (*N* = 6) and adoptive transfer of RUPP CD4+ T cells into NP rats. Mean ± SEM. * *p* < 0.05.

Although it is not fully understood where it is that activated T cells hone during PE, it has been demonstrated by LaMarca’s research group that adoptive transfer of CD4+ cells from RUPP rats into normal pregnant rats elicits the onset of hypertension and reduces GFR suggesting that they target the kidney [134] (Figure 8). This immune cell type has been shown to promote increases in blood pressure in numerous models of hypertension as well [135,136,137]. Here T cells have been shown to infiltrate into the kidney, and they have the capability to produce ROS and angiotensin II [136,138]. Activated immune cells are potent sources of cytokines like TNF-α and IL-6 [128]. TNF-α is able to increase signaling components of the renin-angiotensin system, such as AT1R receptor expression, *in vitro* in cardiac fibroblasts [139]. Importantly, TNF-α-induced hypertension in pregnant rats is significantly attenuated by the AT1R antagonist losartan [140].

The cytokines TNF-α, IL-6 and IL-17 can themselves produce hypertension when infused into pregnant rats. Each of these cytokines is produced by activated CD4^+^ T cells in response to placental ischemia [23,141,142,143,144]. Indeed, culturing of CD4^+^ T cells isolated from RUPP rats on gestational day 19 demonstrated that these cells do release greater amounts of TNFα, IL-6 and IL17 into their culture media. With regards to obesity, it was shown that feeding 4-week-old C57BL/6J mice a 40% high-fat diet consisting compared to the control group receiving a control diet having 14% fat had significantly increase body weight with increased area under the curve for both glucose and insulin tolerance tests [145]. At GD 18.5, body weight and fasting glucose, insulin, triglycerides and total cholesterol were greater in the high-fat diet group. This was accompanied by approximate 9× and 3.5× increases in serum levels of TNFα and IL-1β, respectively. Furthermore, in the placentas from the high-fat diet mice, there were increased mRNA levels of TNFα and IL-6, and immunohistochemistry revealed that NF-kB and phosphorylated NF-kB levels were also up. NF-κB is a transcription factor, that when phosphorylated, locates to the nucleus to positively regulate pro-inflammatory gene expression [146,147]. Importantly, this study found that these inflammatory changes were accompanied by increased placental hypoxia, as detected by HIF-1α mRNA levels. Collectively, these data suggest that obesity and its comorbidities including diabetes are deleterious to placental and pregnancy health via pro-inflammatory mechanisms.

Diabetes is a serious complication of obesity, which has been associated with metabolic disturbances such as increased insulin, free fatty acids and glycerol [148,149]. Obese pregnant women have increased risk for gestational diabetes mellitus (GDM) (odds ratio [OR], 2.6; 95% confidence interval [CI], 2.1–3.4) [150]. Those pregnant women who develop GDM are also at greater risk for PE [151]. Messenger RNA expression of leptin and TNF-α are greater in term placenta (38–39 weeks) in patients with GDM over control subjects [152]. In a meta-analysis of 27 trials, it was revealed that maternal circulating concentrations of leptin and TNF-α were greater whereas there was reduced adiponectin, which is an insulin-sensitizing and vasoprotective molecule, in GDM patients versus controls [153]. In an experimental model of new-onset diabetes in pregnancy induced in Wistar rats by intraperitoneal injection of streptozotocin on day 5 of gestation, ionomycin-induced calcium mobilization in T cells was greater from diabetic pregnant rats on gestational days 12 and 21 than control rats [154]. Intriguingly, supplementing these diabetic pregnant rats with a diet rich in unsaturated fat 15 days prior to pregnancy and throughout gestation prevented this enhanced response to ionomycin. As calcium mobilization and calcium-sensing receptor (CaSR)-induced secretion of cytokines like TNF-α is increased in T cells from inflammatory disease such as sepsis [155], we propose that these pathways link obesity and inflammation to the development of PE.

### 4.2. B lymphocytes

B lymphocytes are involved in the humoral arm of the adaptive immune response [156]. This means that, in response to presentation of foreign antigens by antigen presenting cells like macrophages or T cells, B cells are capable of producing massive amounts of antibodies that circulate in the bloodstream to ward off infections. However, in states of autoimmunity, which is thought to occur in the pathogenesis of PE, B cells are able to mount an antibody response to self-antigens [157]. There are numerous theories whereby those individuals with autoimmune diseases escape immune tolerance [158]. Although the mechanisms that allow this to occur during PE are unclear, it has been shown that B cells are capable of promoting the development of placental ischemia-induced hypertension.

Accumulating data have showed that B cells are important downstream effector cells of activated CD4+ T cells during placental ischemia. Pharmacological-induced depletion of B cells using rutiximab significantly attenuated the development of hypertension and AT1-aa levels in RUPP rats [159]. Subsequent studies revealed that B cells are important in the mechanism whereby CD4+ T cells isolated from RUPP rats elicit hypertension when adoptively transferred into normal pregnant recipient rats [134] (Figure 8). The hypertension following this adoptive transfer was inhibited not only by rutiximab but also by losartan. In addition, it was demonstrated in this adoptive transfer study that there was a significant increase serum levels of AT1-aa. AT1-aa is agonistic toward the AT1R receptor, and studies have shown that Ang II-induced hypertension is exaggerated by co-infusion of AT1-aa [160]. AT1-aa levels are elevated in PE women and animal models of PE [23,143,161]. However, the effects of AT1-aa on the development of obesity-induced hypertension during pregnancy are unknown.

Intriguingly, obese pregnant women have a higher proportion of circulating B cells compared to lean pregnant women [162]. Although the levels of AT1-aa have not been examined with relation to obesity in PE, a glimpse into the possibly of this interaction was recently provided in male mice showing that high-fat diet-induced obesity promotes the production of a number of autoantibodies [163]. The AT1-aa levels were not examined per se in that study though, but with regards to the blood pressure response to AT1-aa during obesity in pregnancy, data do suggest that this response maybe exaggerated compared to lean circumstances. Ang II-induced hypertension is exaggerated in male obese versus lean Zucker rats [164]. Moreover, Ang II-induced forearm vasoconstriction is greater in men with upper body obesity [165]. Although these studies were conducted in males, it is known that AngII and AT1-aa produce hypertension when infused into lean pregnant rats and mice [160]. Future studies should examine the hemodynamic responses to AT1-aa in obese pregnant animal models and also the levels of this autoantibody in obese *versus* lean PE women.

## 5. Impact of Obesity on Placental Ischemia-Induced Vascular Inflammation and Dysfunction

As obesity is in its own right a state of chronic inflammation, it would seem that obese pregnant women are primed to have elevated inflammatory responses to placental ischemia. There are higher CD4^+^ cell counts found in non-pregnant, obese women [34]. Although there are limited studies in this area in pregnancy, data do suggest that obese pregnancies have endothelial-dependent and independent dysfunction compared to lean pregnancies. In experimental animal studies, it was demonstrated in rats fed a diet high in fat with 20% lard before and during pregnancy presented at the end of gestation with increased body weight and reduced maximum responsiveness of mesenteric arteries to acetylcholine-induced vasorelaxation [166]. Although inflammation was not examined in this rat study, it has been more recently shown that obese versus lean pregnant women have endothelial dependent and independent vascular dysfunction associated with increased production of inflammatory cytokines like IL-6, C reactive protein, sICAM-1, PAI-1 and PAI-2 [42]. Furthermore, neutrophils, which can be activated by T cells [167], are increased with production of the ROS generator myeloperoxidase around blood vessels in obese pregnant women [168,169].

The mechanisms that promote greater activation of immune cells and vascular dysfunction in obese pregnancies, and whether these pathways are linked to promote exaggerated placental ischemia-induced hypertension during pregnancy are unknown. One mechanism could be the action of specific metabolic factors such as high levels of leptin, cholesterol, fatty acids, insulin or glucose. It has been shown that high glucose levels activate nuclear factor of activated T cells in smooth muscle cells from cerebral arteries from mice [170]. There is impaired endothelial cell Ca^2+^ signaling in uterine radial arteries isolated from pregnant rats having streptozotocin-induced diabetes and hyperglycemia [8]. This is relevant to the finding that elevated HbA(1c) levels are associated with the exaggerated effects of overweight and obesity on hypertensive disorders of pregnancy [171]. Furthermore, it has been shown that the clinical manifestations of PE are preceded by high levels triglycerides [172] and that LDL cholesterol increases with increasing BMI in pregnancy [173]. Vasorelaxation is impaired by hypercholesterolemia during pregnancy [174]. Whether inflammation plays a role in these processes is unclear, but cholesterol lowering in male apoE^−/−^ mice significantly reduced T cell proliferation [175].

As for circulating cytokine levels in obese PE women, the increased TNF-α levels found in the circulation of lean PE women have been shown not to be exaggerated in obese PE [175]. However, local levels of TNF-α, IL-6 and IL-1 in the placenta are greater in women with obesity compared to healthy controls [176]. Linkage and association studies found in a French-Canadian population that the TNF-α gene locus appears to most significantly influence accumulation of fat in the thigh region of women than men [177]. Future studies should examine local production of cytokines from immune cells, placenta, blood vessels and adipose tissue. Furthermore, the biological activity of cytokines that are increased during placental ischemia should be examined and whether this activity is exaggerated by obesity or specific metabolic factors such as high levels of leptin, cholesterol, fatty acids, insulin or glucose.

Another important mechanism whereby obesity could enhance the renal hemodynamic and blood pressure responses to placental ischemia in pregnant rats is by increasing placental production of the novel anti-angiogenic factor, sFlt-1. This factor binds and quenches the bioactivity of vascular endothelial growth factor (VEGF), which is important for healthy endothelial function [178,179]. This factor is also produced by T cells in response to placental ischemia [128]. Several lines of evidence support the hypothesis that sFlt-1 contributes to maternal endothelial cell activation/dysfunction by antagonizing the angiogenic factors VEGF and PlGF [180,181,182,183,184,185]. Several clinical studies have reported high serum sFlt-1 and low serum free PlGF and free VEGF in PE women [180,181,182,183]. We have repeatedly shown that RUPP in pregnant rats increases plasma and placental levels of sFlt-1 and decreases circulating free VEGF and PlGF levels [23,186,187]. We reported that the placenta is a source of this sFlt-1 because hypoxia solicited placental secretion of sFlt-1 *in vitro* [188,189]. While circulating levels of angiogenic/anti-angiogenic factors have been well characterized in PE, studies in obese PE women are limited [190,191,192,193]. The only available data are indirect studies reporting that the levels of free PlGF are lowest in obese PE women, which suggests higher levels of sFlt-1 [190]. As a caveat, some studies have found sFlt-1 to be lower in obese versus lean PE women [194]. However, whether obese PE women have greater sensitivity to greater endothelial dysfunction induced by soluble placental factors such as sFlt-1, TNF-α or AT1-aa should be investigated. It is suggested that this is the case as obese PE women with reduced sFlt-1 also have reduced adiponectin, which is a vasoprotective adipokine [195].

## 6. ET-1 as a Mechanism Linking Obesity, Inflammation and Hypertension in PE

ET-1 is the most potent vasoconstrictor known [196]. It is a 21-amino acid peptide produced by cleavage from its precursor prepro-ET. ET-1 acts on two receptors: ET_A_ and ET_B_. ET_A_ receptors are predominately located on vascular smooth muscle cells where they mediate vasoconstriction by ET-1 produced from the adjacent endothelial cells. In contrast, this vasoconstriction is balanced by ET_B_ receptors located on the endothelium that mediate vasorelaxation through NO-mediated mechanisms. ET-1 is elevated in PE women and animals models of PE [197].

ET-1 plays an important role in mediating the hypertension in response to RUPP. Indeed, chronic administration of an ET_A_ receptor antagonist completely abrogated the increase in MAP in RUPP rats [198,199]. Moreover, TNF-α mediates the renal and blood pressure responses to RUPP via activation of the ET-1 system [142,143]. Similar findings were found regarding the hypertensive response to chronic TNF-α excess in pregnant rats [24]. Intriguingly, TNF-α levels are the greatest in placental tissue from obese PE women [114]. Thus, exacerbation of TNF-α-induced ET-1 synthesis could be one mechanism whereby obesity or specific metabolic factors may amplify the arterial pressure response to RUPP.

A direct role for AT1-aa in mediating hypertension during pregnancy via ET-1 was revealed following infusion of purified AT1-aa into pregnant rats from GD 12–19; this protocol increased renal levels of preproET-1 mRNA and MAP, and both of these responses were attenuated by oral administration of an ET_A_ antagonist [161]. AT1-aa can directly stimulate ET-1 in cultured human umbilical vein endothelial cells [141,143,144,161]. While these findings indicate an important interaction between AT1-aa and the ET-1 system in the response to placental ischemia, the effect of obesity or obesity-related metabolic factors such as leptin on AT1-aa-induced ET-1 production has not been examined.

Convincing evidence from our laboratory and others support a role for sFlt-1 in the pathophysiology of hypertension in response to placental ischemia [23,186,187,200,201]. Chronic infusion of sFlt-1 from GD 14–19, at a rate to mimic the circulating levels observed in PE women and RUPP rats, produces hypertension in normal pregnant rats [200,201]. This increase in plasma sFlt-1, which significantly decreased plasma bioavailable VEGF, was associated with a 20 mmHg rise in MAP with decreased GFR [200,201]. There is strong evidence that this reduction in renal hemodynamics first results from decreased VEGF leading to reduced vasodilators like NO and increased levels of the vasoconstrictor ET-1 in microvascular endothelial cells [202,203]. Indeed, sFlt-1-induced hypertension in pregnant rats markedly elevated renal cortical ET-1 levels [204]. Separate complementary studies showed that a selective ET_A_ antagonist completely blocked sFlt-1-induced hypertension in pregnant rats. However, while administration of sFlt-1 to pregnant rats causes hypertension, it is unknown whether obesity or metabolic derangements such as hyperleptinemia enhances the blood pressure and renal responses to sFlt-1 during pregnancy.

Leptin plays a critical role in the hypertension associated with obesity, and several studies suggest that this is tied to activation of the ET-1 system via increased signaling at the ET_A_ receptor [205,206,207,208,209,210]. *In vitro* evidence has demonstrated that treatment of human umbilical vein endothelial cells with obesity-related levels of leptin (100 ng/mL) greatly increased expression of ET-1 compared to those levels encountered in normal weight humans (~10 ng/mL) [211]. *In vivo* data from Hall and colleagues demonstrated that high-fat diet-induced obesity in male rats produced increases in circulating leptin by 3× along with hypertension, and this blood pressure response was significantly attenuated by an ET_A_ antagonist [212]. Intriguingly, leptin levels are found to be the highest in obese PE women compared to either co-morbidity alone, and there is a correlation between leptin and sFlt-1 levels in PE but not normotensive pregnant women [213]. However, there have been no studies directly linking these two factors to the development of hypertension and whether this is mediated by ET-1.

It is important to mention that there are two clinical subtypes of PE termed early- and late-onset. Both are associated with increased maternal death compared to women without either subtype [214], however they are thought to mediate the development of PE via differing mechanisms. Deficient spiral artery remodeling is hypothesized to account for the majority of the pathogenesis of the early-onset subtype of PE. Early-onset PE usually presents itself before 34 weeks of gestation [215]. Early-onset PE is less common overall, and as Figure 1 indicates, the majority of obese patients suffer from the late-onset disease, which occurs after 34 weeks. In fact, the incidence of early-onset PE remains similar across all obesity classes (0.3%–0.4%), whilst the incidence of the late-onset disease increases in an upward trend as the severity of obesity increases (from 7.4% in class I to 13% in super-obese patients). Spiral artery remodeling is not always perturbed in the late-onset PE, which is also sometimes referred to as maternal PE as it is thought that the mother cannot meet the metabolic demands of the enlarged placenta leading eventually to placental hypoxia [216]. The fact that the majority of obese patients are much more likely to develop the late-onset disease is likely to be related to the increased blood pressure responsiveness of these patients to placentally-derived cytokines and anti-angiogenic factors. This increased sensitivity is likely related to cardiovascular dysfunction occurring prior to pregnancy in obese women, such as underlying inflammation, metabolic syndrome and high levels of adverse adipokines. The fact that there is an increased trend in the incidence of the late-onset disease suggests that there in a strong interaction between maternal cardiovascular dysfunction and placental hypoxia linking obesity to the development of PE.

## 7. Summary of Proposed Mechanisms and Conclusions

**Figure 9:**
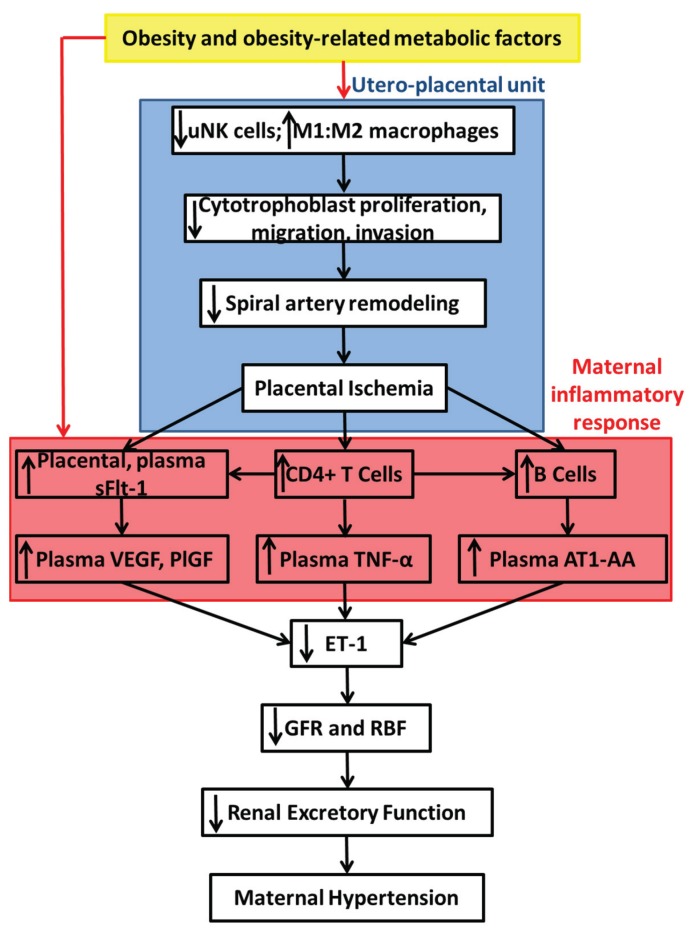
A hypothetic scheme proposing sites where obesity and obesity-related metabolic factors such as high levels of leptin, cholesterol, fatty acids, insulin and/or glucose exaggerate the cascade of events in the utero-placental unit leading to the development of placental ischemia (highlighted by the blue box) and placental ischemia-induced maternal inflammatory responses (highlighted by the red box) culminating in an augmented placental ischemia-induced maternal hypertensive response in obesity.

This review focused on the immune basis of PE. It has been shown that obesity and its related metabolic factors activate the immune system, which potentially explains the link between obesity and increased risk for this maternal disorder. However, this has been studied mostly under non-pregnant conditions in humans and animal models. Therefore, it is unclear how obesity impacts on immune mechanisms involved in the cascade of events leading to placental ischemia-induced hypertension. We propose that activation of the maternal innate and adaptive immune systems may mediate the mechanisms whereby obesity promotes the development of placental ischemia. Furthermore, Figure 9 highlights a hypothetical scheme detailing mechanisms whereby we believe obesity and obesity-related metabolic factors may act to exaggerate the development of placental ischemia-induced hypertension. Studies linking obesity-related metabolic factors, inflammation, and hypertension and vascular dysfunction in PE are warranted. Such studies may help basic science researchers and clinicians better understand the etiology of PE.
